# Enabling Self-passivation by Attaching Small Grains on Surfaces of Large Grains toward High-Performance Perovskite LEDs

**DOI:** 10.1016/j.isci.2019.07.044

**Published:** 2019-07-31

**Authors:** Jiajun Qin, Jia Zhang, Yujie Bai, Shengbo Ma, Miaosheng Wang, Hengxing Xu, Matthew Loyd, Yiqiang Zhan, Xiaoyuan Hou, Bin Hu

**Affiliations:** 1 Department of Materials Science and Engineering, University of Tennessee, Knoxville, TN 37996, USA; 2 State Key Laboratory of Surface Physics, Key Laboratory of Micro and Nano Photonic Structures (Ministry of Education) and Collaborative Innovation Center of Advanced Microstructures, Fudan University, Shanghai 200433, China; 3 Center for Micro Nano System, SIST, Fudan University, 200433 Shanghai, China

**Keywords:** Materials Characterization, Optical Materials, Surface

## Abstract

This paper reports a new method to generate stable and high-brightness electroluminescence (EL) by subsequently growing large/small grains at micro/nano scales with the configuration of attaching small grains on the surfaces of large grains in perovskite (MAPbBr_3_) films by mixing two precursor solutions (PbBr_2_ + MABr and Pb(Ac)_2_·3H_2_O + MABr). Consequently, the small and large grains serve, respectively, as passivation agents and light-emitting centers, enabling self-passivation on the defects located on the surfaces of light-emitting large grains. Furthermore, the light-emitting states become linearly polarized with maximal polarization of 30.8%, demonstrating a very stable light emission (49,119 cd/m^2^ with EQE = 11.31%) and a lower turn-on bias (1.9 V) than the bandgap (2.25V) in the perovskite LEDs (ITO/PEDOT:PSS/MAPbBr_3_/TPBi[50 nm]/LiF[0.7 nm]/Ag). Therefore, mixing large/small grains with the configuration of attaching small grains on the surfaces of large grains by mixing two precursor solutions presents a new strategy to develop high-performance perovskite LEDs.

## Highlights

• Small grains are attached to the surfaces of large grains in perovskite films
• Ions can passivate grain boundary defects to enable self-passivation in PeLEDs
• Light-emitting states become linearly polarized upon self-passivation
• Turn-on voltage is lower than bandgap in perovskite LEDs under self-passivation

## Graphical Abstract

**Figure:**
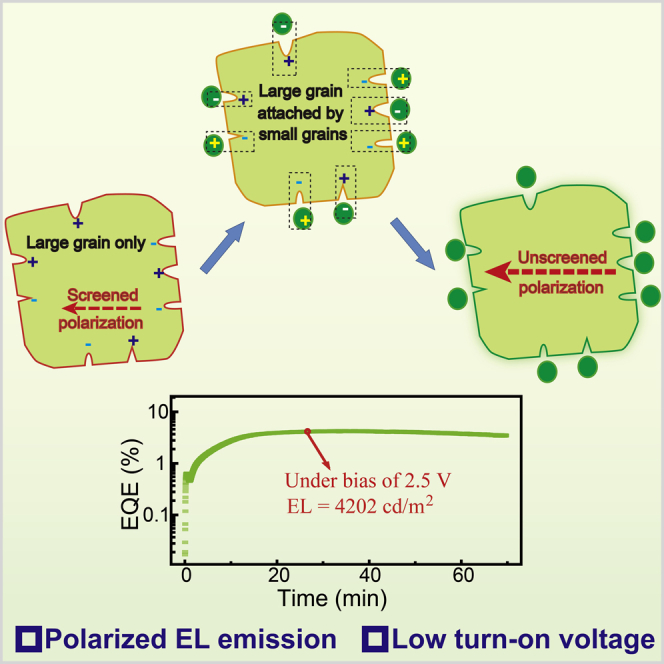

## Introduction

Organic metal halide perovskites have become attractive candidates to develop thin-film light-emitting devices (LEDs) with low turn-on voltage (Wang et al., 2015, Meng et al., 2017), ambipolar transport (Shi et al., 2015, Stranks et al., 2013), large color-tuning properties (Xing et al., 2014, Tan et al., 2014, Protesescu et al., 2015), and high device efficiencies (Kim et al., 2015, Li et al., 2015, Xing et al., 2016, Yu et al., 2016, Ning et al., 2015, Wang et al., 2016a, Deschler et al., 2014, Sutherland and Sargent, 2016). The external quantum efficiencies (EQE) have been quickly improved from 0.1% to over 20% based on the effort from materials processing and device-interface engineering (Tan et al., 2014, Wang et al., 2016a, Yang et al., 2018, Cao et al., 2018, Lin et al., 2018, Zhao et al., 2018). It is noted that polycrystalline perovskites are often formed with a large amount of grain boundary defects, which can cause serious non-radiative recombination (Nie et al., 2015, Hsiao et al., 2015). Therefore, controlling grain boundary defects becomes a critical procedure to develop highly efficient perovskite LEDs. In general, there are two different ways to control grain boundary defects: (1) extrinsic method through doping or thermal/solvent treatment (Hadadian et al., 2016, Wang et al., 2016b) and (2) intrinsic method by using self-passivation during device operation (Bi et al., 2017). In particular, organic cations can easily become mobile ions under electrical field due to low potential barriers, ionically quenching light-emitting states in organic-inorganic hybrid perovskites (Yuan and Huang, 2016, Chen et al., 2015). The recent studies have shown an extrinsic approach to constrain the mobile ions by introducing polymer chains into polycrystalline perovskites (FAPbBr_3_), which consequently leads to a high electroluminescence (EL) efficiency (14.36%) (Yang et al., 2018). Clearly, controlling mobile ions becomes a critical procedure to develop high-performance perovskite LEDs.

Our early studies have shown that mobile ions can change local semiconducting properties in hybrid perovskite single crystals (MAPbI_3_) when they are driven by an electrical field (Wu et al., 2017a). This observation brings an open question on whether mobile ions can be used as dopants to passivate grain boundary defects in perovskite LEDs. Toward understanding the effects of ions on light emission, it was shown that the PL can be enhanced in the perovskite (MAPbBr_3_) single crystal when mobile ions (Br^−^) are driven to light-emitting locations by an electrical field between two lateral electrodes (Luo et al., 2017). Although this occurs in perovskite single crystal, it provides a further promising information for polycrystalline perovskite thin-film devices to support the argument that mobile ions can be used as dopants to decrease the grain boundary defects. In this work, we introduce a new method to enable the self-passivation of grain boundary defects to develop stable and high-brightness EL by using subsequent growth of large and small grains with the configuration of attaching small grains on the surfaces of large grains based on one-step solution procedure. Our method utilizes the mixture of two precursor solutions, lead bromide (PbBr_2_) and lead acetate trihydrate (Pb(Ac)_2_·3H_2_O) solutions, to subsequently grow large and small grains with micro- and nano-scales based on fast and slow crystallization rates enabled by solution concentrations and solvent polarities (Bai et al., 2019). Essentially, subsequent growth of large and small grains forms the unique configuration of attaching small grains on the surfaces of large grains, leading to small and large grains as dopants and light-emitting centers, respectively. Specifically, spin-casting induces the fast formation of large grains from the PbBr_2_ source due to high crystallization rate, pushing Pb(Ac)_2_·3H_2_O source to the grain boundaries in the liquid containing two precursor solutions (PbBr_2_ + MABr and Pb(Ac)_2_·3H_2_O + MABr). Upon applying thermal annealing, the residual Pb(Ac)_2_·3H_2_O source grows into small grains on the surfaces of large grains to form mixed large/small grains with high/low bandgap. This design intends to serve three purposes. First, attaching the high-bandgap small grains to the surfaces of low-bandgap large grains forms interfacial potential wells that can spatially confine grain boundary defects and mobile ions to enable self-passivation during device operation. Second, the small grains with nanometers can effectively confine injected electrons and holes and then efficiently cascade the injected carriers from high-bandgap small grains to low-bandgap large grains functioning as light-emitting centers. Third, attaching small grains to the surfaces of large grains leads to high-quality films for device fabrication. With this design, the self-passivation can be realized when mobile ions are electrically driven to grain boundaries to neutralize the charged defects. As a result, this design presents a new method to develop high-performance perovskite (MAPbBr_3_) LEDs with very stable and high-brightness EL by enabling the self-passivation.

## Results and Discussion

### Morphological Structures of Mixed Large/Small Grains

**Figure 1:**
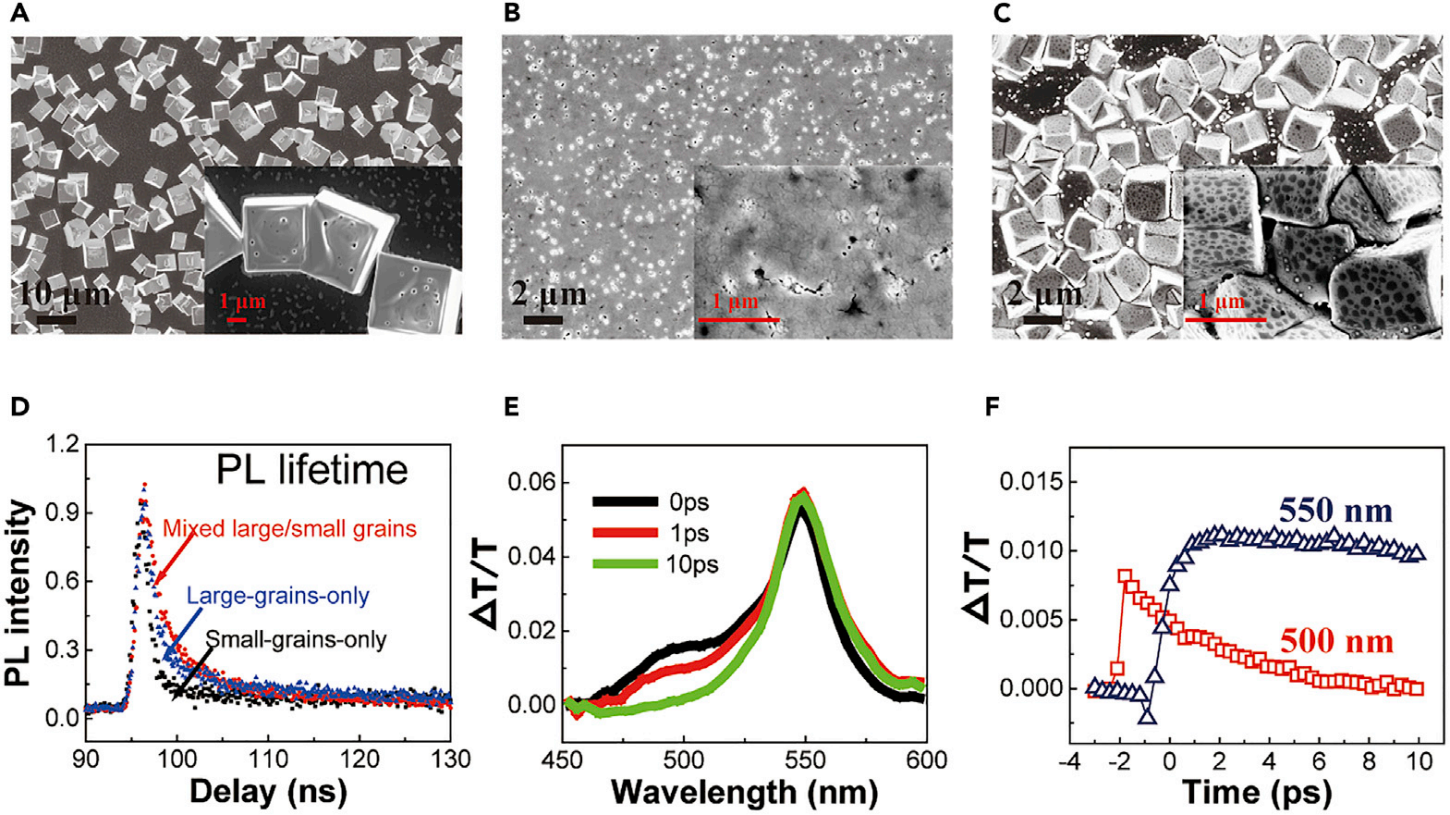
SEM Images to Show Hybrid Perovskite (MAPbBr_3_) Film Morphologies together with PL and TA Dynamics (A) SEM for large-grains-only film (see also Figure S1B). (B) SEM image for small-grains-only film (see also Figure S1A). (C) SEM image for mixed large/small grains (see also Figure S1C). (D) PL lifetimes for mixed large/small grains, small-grains-only, and large-grains-only films. (E) TA spectra measured at 0, 1, and 10 ps. (F) TA lifetimes at 550 and 500 nm corresponding to large-grains and small-grains components. See also Figures S1 and S2.

In the precursor containing PbBr_2_ and MABr with DMF as solvent, these two strong electrolytes can dissociate into Pb^2+^, MA^+^, and Br^−^ ions, enabling very fast crystallization to quickly grow large grains as large as several micrometers during spin-coating before thermal annealing, indicated by the scanning electron microscope (SEM) image in Figure 1A and atomic force microscopy (AFM) images (see Figure S1B). On contrast, in the precursor containing Pb(Ac)_2_·3H_2_O and MABr in DMF, Pb(Ac)_2_·3H_2_O, known as weak electrolyte, can partially dissociate into Pb^2+^ ions with very low concentration, resulting in the growth of small grains due to slow crystallization rate. During the annealing process, Pb(Ac)_2_·3H_2_O can release Pb^2+^ ions for crystal growth (see Figure 1B with grain size <100 nm and Figure S1A). By mixing these two precursor solutions through a one-step processing procedure, the ions (Pb^2+^, MA^+^ and Br^−^) tend to aggregate as the solvent is evaporated during spin-casting, leading to the fast formation of large grains. As the large grains are grown, the Pb(Ac)_2_·3H_2_O molecules are driven to the grain boundaries. During thermal annealing, the Pb(Ac)_2_·3H_2_O-based source starts to release Pb^2+^ ions for the crystallization of MAPbBr_3_ on the boundaries of large grains. Therefore, subsequently growing large and small grains with micro- and nano-scales by mixing two precursor solutions forms unique configuration of attaching small grains on the surfaces of large grains to prepare perovskite (MAPbBr_3_) films with mixed large/small grains through this simple one-step solution processing method, enabling self-passivation ready to develop high-performance perovskite LEDs. The SEM image in Figure 1C provides a direct evidence to verify that the small grains with the size of around 100 nm are indeed attached to the surfaces of large grains with the size of 1–2 μm to form mixed large/small grains in the hybrid perovskite (MAPbBr_3_) films (see also Figure 1C). According to X-ray diffraction patterns (Figure S2), all three films show a dominant peak at around 14.8^0^, which corresponds to the (1 0 0) cubic face of MAPbBr_3_ crystal. No residual acetate is observed in the small-grains-only film. We should mention that our design of mixing large and small grains can generate high-quality perovskite films ready for fabricating perovskite LEDs. On contrast, the use of only large or small grains often encounters a difficulty to prepare high-quality perovskite films due to the undesirable pinholes. We can see in Figure 1D that the perovskite film with mixed large/small grains presents a longer PL lifetime (τ = 3.11 ns) as compared with small-grains only (τ = 1.35 ns) and large-grains only (τ = 1.87 ns) films. Here, the longer PL lifetime observed from mixed large/small grains provides an evidence that attaching small grains to the surfaces of large grains introduces an interaction between large- and small-grain components toward self-passivation. We can see in Figure 1E that the transient absorption (TA) at 500 nm quickly decreases within 10 ps in the hybrid perovskite films with mixed large/small grains. The positive TA signal in Figures 1E and 1F corresponds to the PL emission. Based on our PL results, the signals at 500 and 550 nm are the emission from small-grain and large-grain components, respectively. Owing to bandgap offset between small grains (wide bandgap) and large grains (narrow bandgap), carrier transfer from small grains to large grains can occur. As a result, the TA signal from small grains (500 nm) ceases quickly (<6 ps), whereas the TA signal from large grains (550 nm) does not change within 10 ps. Moreover, this carrier transfer is further evidenced by the EL spectrum observed only from large-grain components because all the injected carriers are cascaded to large grains for recombination in the mixed large/small grains. As the TA signal in the small-grains component gradually decreases, the TA signal from large grains (at 550 nm) is established in the hybrid perovskite films. This TA dynamics indicates an efficient charge transfer between large and small grains, providing the precondition toward self-passivation to neutralize the charged defects at the boundaries in mixed large/small grains.

### Mixing Large/Small Grains to Enable Self-Passivation by Using Ion Migration

**Figure 2:**
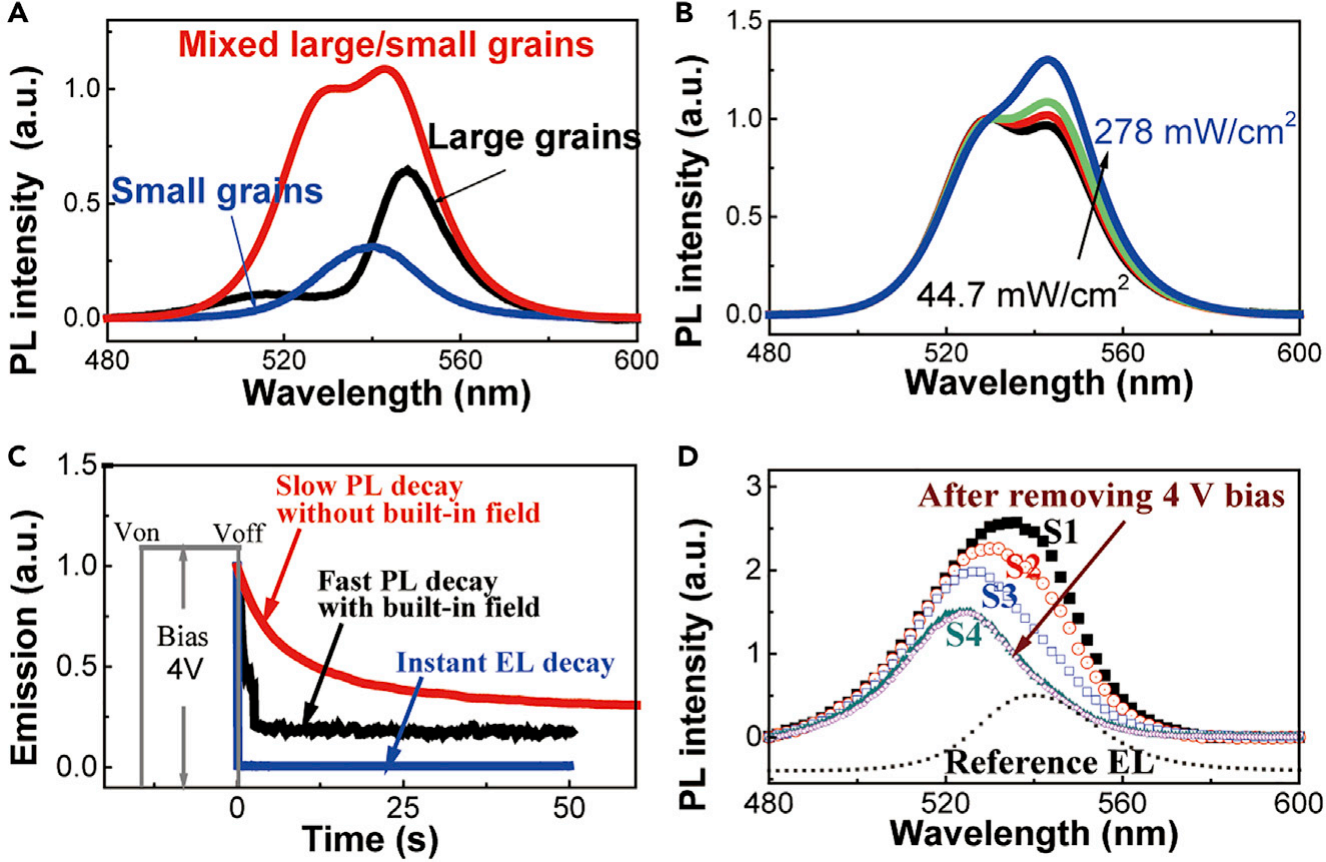
PL Studies on Self-passivation Mechanism (A) PL spectra for MAPbBr_3_ films with mixed large/small grains, small grains-only, and large grains-only. (B) PL spectra for mixed large/small grains under different photo-excitation intensities: 44.7, 117.2, 228, and 278 mW/cm^2^. (C) PL intensity is plotted as a function of time after 4 V bias is removed while keeping constant photoexcitation for two different situations: without and with built-in field. EL intensity is instantly decayed right after the 4 V bias is removed (see also Figure S3). (D) PL involution after removing an electrical excitation (4 V) while keeping constant photoexcitation (405 nm and 200 mW/cm^2^). PL spectrum is gradually changed from S1 (t ∼ 0 s) to S2 (t ∼ 20 s), S3 (t ∼ 100 s), and S4 (t > 5 min) after removing the 4 V bias. When a built-in field is established by connecting ITO and Ag contacts after the 4 V bias is removed, the PL spectrum is quickly changed from S1 to S4. See also Figure S3.

Here we discuss the energy transfer from high-bandgap small grains to low-bandgap large grains in the hybrid perovskite films with the configuration of attaching small grains to the surfaces of large grains. Figure 2A shows the PL spectra for perovskite films prepared with mixed large/small grains, small-grains-only, and large-grains-only designs. The small-grains-only and large-grains-only films give rise to different PL peaks at 539 and 548 nm, respectively, which clearly indicate high and low bandgaps. In the mixed large/small grains where small grains are attached to the surfaces of large grains, the two PL peaks are observed at 528 and 546 nm based on the emission from small-grain and large-grain components, which are slightly blue-shifted as compared with the PL peaks (539 and 548 nm) of small-grains-only and large-grains-only films. This difference indicates that the large and small grains in the mixed large/small grains have slightly different physical sizes as compared with the large-grains-only and small-grains-only systems. Figure 2B shows the PL spectra for mixed large/small grains at different photoexcitation intensities. We can see that, with increasing photoexcitation intensity of exciting both large-grain and small-grain components by using the laser beam of 405 nm, the large-grain and small-grain components become a dominant light-emitting center at high and low excitation intensities in the mixed large/small grains. This observation provides two important information. First, there exists a cascade phenomenon for energy transfer from the small-grain component to the large-grain component, indicating a band offset between large-grain and small-grain components in the mixed large/small grains. This band offset provides a precondition to form interfacial potential wells at large/small grain boundaries. Second, when the small-grain component with high bandgap functions as capture center to confine electrons and holes, the confined electrons and holes can cascade into the large-grain component to give rise to a light emission in the mixed large/small grains.

Now, we use electrically modulated PL studies to explore the self-passivation on grain boundary defects from electrically driven ions during the EL operation in the perovskite LEDs prepared with mixed large/small grains. In the electrically modulated PL, both PL and EL are generated in the perovskite LED made from mixed large/small grains by simultaneously applying photoexcitation (405 nm laser with intensity of 200 mW/cm^2^) and electrical excitation (4 V bias), as shown in Figures 2C and 2D. After removing this 4 V bias, the EL immediately disappears, leaving the PL as the only light emission at constant photoexcitation. Interestingly, the PL intensity is slowly decreasing in the time range of 1 min after removing the electrical excitation and the EL. Furthermore, after an electrical poling from 4 V bias, with immediately applied photoexcitation the PL shows a slow decay (see Figure S3). This means that removing the applied bias can release the mobile ions from grain boundaries and thus causes a decrease on PL intensity. We can see in Figure 2D that the PL gradually changes from large-grains-dominated emission to small-grains-dominated emission after removing the 4 V bias. Clearly, this electrically modulated PL result indicates that electrically driven ions can decrease the defects on large grains, enabling a self-passivation, and consequently increasing the PL contributed from large-grain component in the mixed large/small grains. Essentially, the self-passivation occurs on the surfaces of large grains in the mixed large/small grains when the small grains are attached to the surfaces of large grains. Here, attaching high-bandgap small grains to the surfaces of low-bandgap large grains provides interfacial potential wells at large/small grain boundaries to spatially confine ions and defects during device operation, enabling a self-passivation.

We should note that ion migration normally quenches light-emitting states in organic-inorganic hybrid perovskites (Chen et al., 2017, Galisteo-López et al., 2016, Hoke et al., 2015). Recently, by using perovskite single crystal (MAPbI_3_), we found that the organic ions can indeed function as dopants to change the semiconducting properties (Wang et al., 2016b). Here, by using thin-film perovskite LEDs we demonstrate that electrically driven ions enable a self-passivation mechanism on grain boundary defects during EL operation in the perovskite MAPbBr_3_ device with mixed large/small grains. Essentially, the self-passivation requires that electrically driven ions and grain boundary defects are spatially confined in the interfacial potential wells at large/small grain boundaries in perovskite films prepared with mixed large/small grains where small grains are attached to the surfaces of large grains.

We further confirm the self-passivation mechanism from electrically driven ions by using device built-in field to control the ion migration in PL measurements. The device built-in field can be established by externally connecting the ITO and Ag contacts in the ITO/PEDOT:PSS/MAPbBr_3_/TPBi(50 nm)/LiF(0.7 nm)/Ag device. The built-in field is essentially generated by the contact potential between ITO and Ag through conducting connection due to different work functions (ITO = 4.7 eV and Ag = 4.4 eV) (Liao et al., 2016). Theoretically, the work function difference between ITO and Ag gives the contact potential of 0.3 V, forming a substantial built-in field. After removing the forward bias while keeping the constant photoexcitation, introducing this built-in field by connecting the ITO and Ag contacts can more quickly release the ions used for self-passivation, causing a quick decay on light emission intensity. Figure 2D shows that the PL is quickly decayed from S1 to S4 when the built-in field is introduced by connecting the ITO and Ag contacts after removing the 4 V bias under the constant photoexcitation. On contrast, without introducing this built-in field, the PL is slowly decayed when the ITO and Ag contacts are un-connected after removing the 4 V bias. Clearly, this quick PL decay caused by the built-in field provides the further evidence to support the self-passivation mechanism from electrically driven ions in the perovskite MAPbBr_3_ LEDs prepared with mixed large/small grains. Here, by monitoring PL intensity (at constant photoexcitation intensity) with and without a built-in field, we found that, under external bias, the mobile ions are essentially passivating the defects, increasing light emission. This is why the PL intensity is fast decreasing at constant photoexcitation by keeping the built-in field. This means that the mobile ions, previously driven to the potential wells at the interfaces between large/small grains under applied bias, are escaping from the large/small grain interfaces, decreasing the passivation effects and causing a decrease on PL intensity at constant photoexcitation. The recent PL studies indicate that ion migration can function as dopants to passivate defects in single crystal perovskite (MAPbBr_3_) with two lateral electrical contacts to drift ion migration (Nie et al., 2015). Here, we use thin-film perovskite LEDs with mixed large/small grains to demonstrate that electrically driven ions can initiate self-passivation, forming the necessary condition to develop high-performance perovskite LEDs at high injection current.

**Figure 3:**
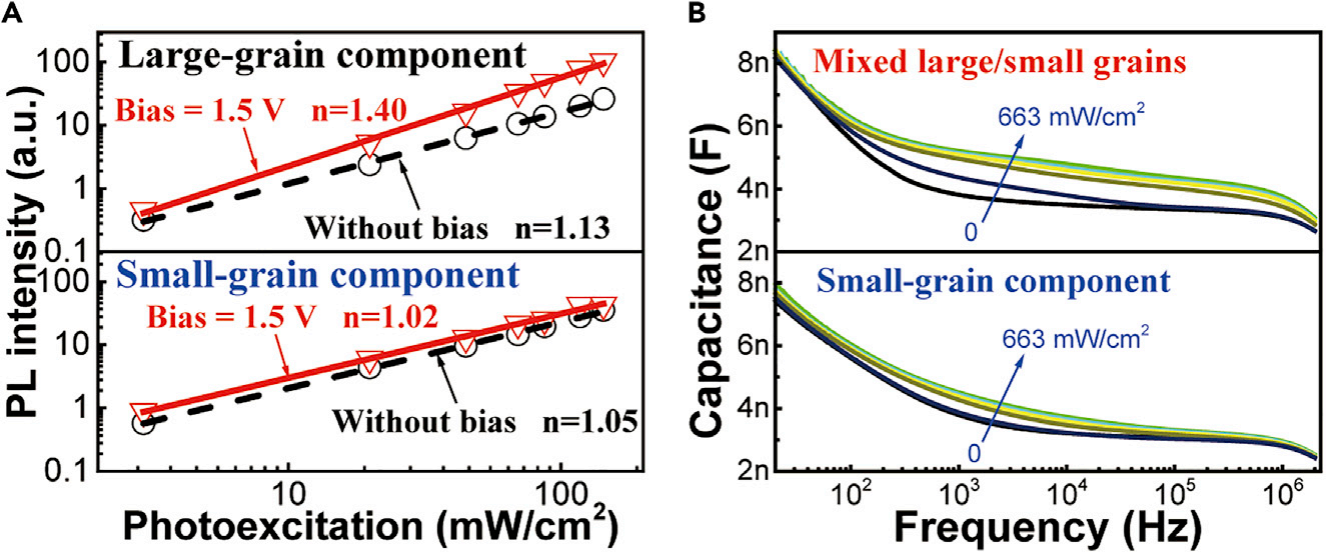
Self-passivation Enabled by Mixing Large/Small Grains (A) PL intensity-photoexcitation intensity curves for large-grain component and small-grain component with/without 1.5 V bias in the perovskite device with mixed large/small grains. (B) Capacitance-frequency characteristics under different photoexcitation intensities: 0, 2.9, 87, 228, 278, and 663 mW/cm^2^. Device structure: ITO/PEDOT:PSS/MAPbBr_3_/TPBi(50 nm)/LiF(0.7 nm)/Ag.

Figure 3A shows the PL intensity as a function of photoexcitation intensity for large- and small-grain components in the mixed large/small grains upon self-passivation under an applied bias of 1.5 V (lower than the EL turn-on voltage of 1.9 V). Applying 1.5 V bias increases the PL-excitation slope from 1.13 to 1.40 for the large-grain component but has a negligible effect on the small-grain component. This further indicates that the self-passivation is enabled to physically passivate the grain boundary defects on the low-bandgap large grains in the mixed large/small grains. Figure 3B shows the capacitance-frequency characteristics at different photoexcitation intensities for small-grains only and mixed large/small grains films. We can see in mixed large/small grains that increasing photoexcitation causes an increase in the capacitance at the dipolar polarization frequency (∼10^5^ Hz). This result suggests that a photoexcitation increases the bulk polarization in dipolar polarization regime in the mixed large/small grains. This presents a further evidence that the grain boundary defects are largely reduced in the mixed large/small grains where small grains are attached to the surfaces of large grains.

### Self-Passivation to Enable Polarization of Light-Emitting States

**Figure 4:**
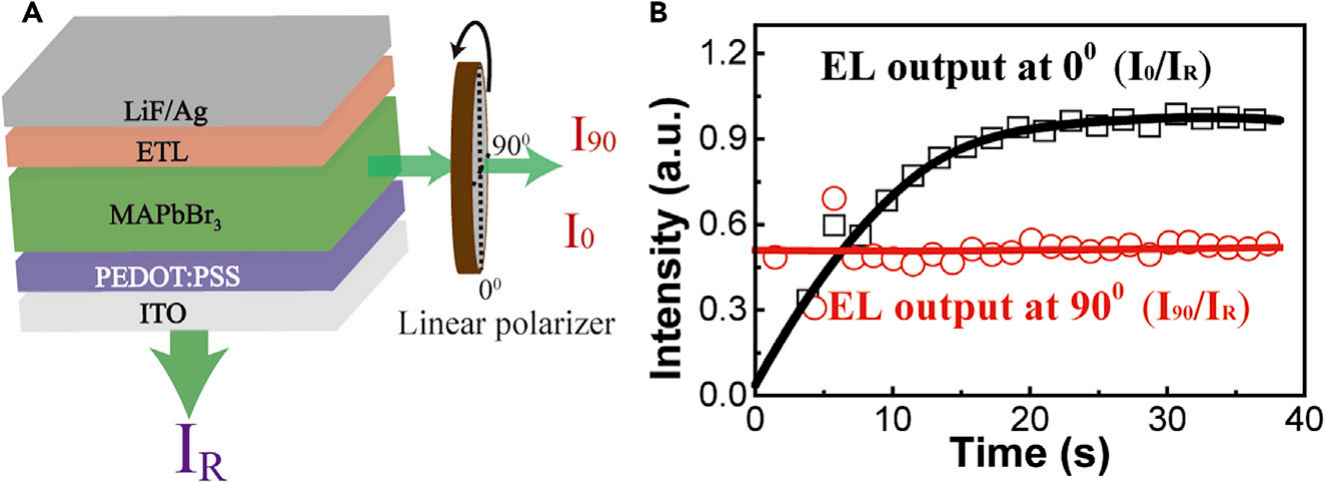
Experimental Results of Polarized Emission under Constant Bias of 3 V (A) Experimental setup to measure EL intensities at 0^°^ and 90^°^ relative to electric field direction. (B) EL outputs (I_0_ and I_90_ relative to reference I_R_) are plotted as a function of time during self-passivation.

Now, we explore the polarization of light-emitting states upon the self-passivation from ion migration in the hybrid perovskites with mixed large/small grains under electrical injection. To detect the polarized output, the EL intensity was simultaneously measured at perpendicular and parallel directions to the device plane, as shown in Figure 4A. The EL at perpendicular direction is used as the reference signal, labeled as **I**_**R**_. The EL at parallel direction, labeled as **I**, is monitored with a linear polarizer set at 0° and 90° relative to electrical field direction, giving **I**_**0**_ and **I**_**90**_. We should note that the EL output is gradually increasing during the self-passivation under given bias. Here, by using the EL output **I**_**R**_ through ITO, we plot the **I**_**0**_/**I**_**R**_ and **I**_**90**_/**I**_**R**_ as a function of time during the self-passivation to reflect the polarization of EL output measured parallel to device plane, as shown in Figure 4B. Very interestingly, we can see that the EL output (**I**_**0**_/**I**_**R**_) measured at 0° to electrical field shows a substantial increase with self-passivation, whereas the EL output (**I**_**90**_/**I**_**R**_) measured at 90° to the electrical field direction is unchanged during the self-passivation. This observation provides a clear evidence that, upon self-passivation, the light-emitting states become linearly polarized to electrical field direction in perovskite LEDs prepared with mixed large/small grains. On contrast, both small-grains-only and large-grains-only devices do not show any polarized EL during device operation.

### Polarizing Light-Emitting States to Generate High-Performance EL

**Figure 5:**
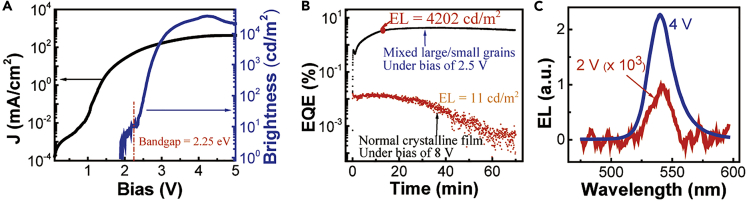
EL Performance for Device with Mixed Large/Small Grains (ITO/PEDOT:PSS/MAPbBr3/TPBi[50 nm]/LiF[0.7 nm]/Ag) (A) EL-current-voltage characteristics (see also Figure S4). (B) EQE as a function of time under constant bias for perovskite LEDs with mixed large/small grains (blue curve) and normal perovskite film prepared by the vapor-assisted two-step method (black curve). (C) EL spectra measured at 2.0 V (slightly above the turn-on bias 1.9 V) and 4 V. See also Figure S4.

**Figure 6:**
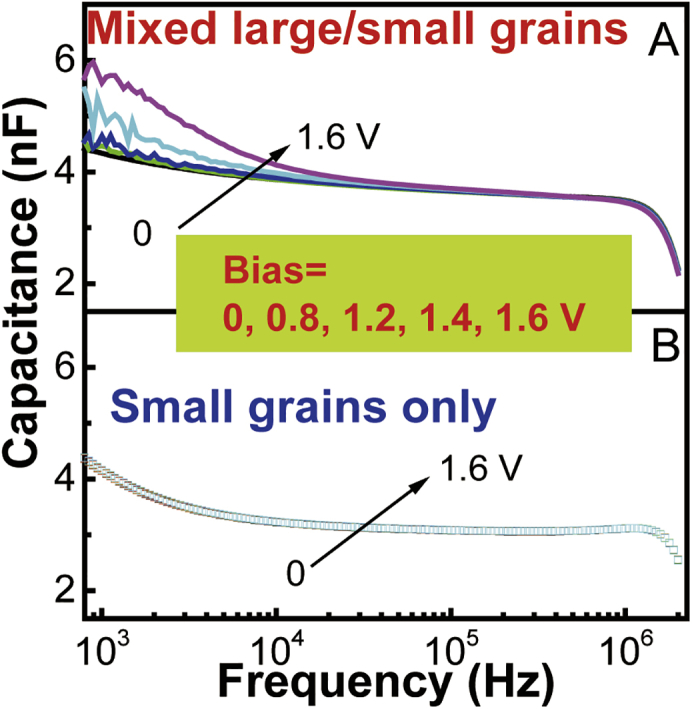
Capacitance-Frequency Curves under Different Bias Device with (A) mixed large/small grains and (B) with small grains only. (Device structure: ITO/PEDOT:PSS/MAPbBr_3_/TPBi[50 nm]/LiF[0.7 nm]/Ag).

Figure 5A presents the high-performance EL for mixed large/small grains with the device architecture of ITO/PEDOT:PSS/MAPbBr_3_/TPBi(50 nm)/LiF(0.7 nm)/Ag upon self-passivation. The device demonstrates a high EL brightness of 37,017 cd/m^2^ at the current density of 397 mA/cm^2^. The maximal efficiency reached 12.7 cd/A. We should note that the current efficiency is continuously increasing without roll-off phenomenon from 200 to 400 mA/cm^2^. Especially, the device shows a turn-on voltage of 1.9 V, lower than the bandgap of 2.25 eV, to activate the EL. It should be noted that using the Bphen and PMMA composite as the electron-transport layer can indeed enhance the self-passivation enabled by electrically driven ions in the perovskite layer. Consequently, the maximal EQE can reach 11.31% without current efficiency roll-off in the perovskite LED (ITO/PEDOT:PSS/MAPbBr_3_/Bphen[20 nm]/Bphen:PMMA/LiF[0.7 nm]/Ag) (see Figure S4). It should be noted that both large-grains-only and small-grains-only perovskite films suffer from poor film quality, leading to unstable LEDs in our studies. Therefore, we compare EL stabilities for the devices made from our mixed large/small grains and the normal polycrystalline perovskite film prepared by vapor-assisted two-step method in Figure 5B. We can see that, the EQE does not show any appreciable decay within 1 hour at the EL brightness of >1,000 cd/m^2^ by using the mixed large/small grains, whereas the device made from normal perovskite film shows an appreciable decay. Here, the EL spectrum in Figure 5C shows only one dominant peak at 540 nm, indicating the emission from large-grain component in the perovskite films prepared with mixed large/small grains. In the EL case, the injected electrons and holes all cascade into large grains for recombination, leading to only one EL peak from the large-grain component in mixed large/small grains. Furthermore, based on the self-passivation occurring in the mixed large/small grains, we can see that the EL can be clearly observed at 2.0 V bias lower than the bandgap of 2.25 eV. A similar phenomenon was experimentally observed in the perovskite FAPbBr_3_ LED (turn-on bias: 1.7 V and bandgap: 2.3 eV) (Meng et al., 2017). The turn-on bias lower than the bandgap was also observed in the LEDs made from CdSe–ZnS core/shell quantum dots (Qian et al., 2011). However, it demands further investigation to reveal the underlying mechanism. Here, we consider that an EL action must satisfy the energy conservation at the turn-on voltage. By considering the energy conservation, the turn-on voltage (V_th_) can be given by

(Equation 1) Eg ≤ e(V_th_ + P_surface_)

where Eg is the bandgap, P_surface_ is the field-induced surface polarization. Clearly, the only possibility of obtaining the turn-on bias lower than the bandgap is to use the P_surface_. Specifically, when the P_surface_ is existed upon self-passivation, the turn-on bias V_th_ can be lower than the bandgap Eg. This is because P_surface_ can function as an additional electrical field to facilitate the electrical injection, leading to the turn-on voltage lower than the bandgap upon self-passivation. Without self-passivation, grain boundary defects can electrically screen the P_surface_, leading to the turn-on voltage larger than the bandgap. By considering the field-induced surface polarization, an EL can be initiated when the applied bias is lower than the bandgap in LEDs. Now, we use Capacitance-frequency (C-f) measurements under dark condition to explore the field-induced surface polarization in the ITO/PEDOT:PSS/MAPbBr_3_/TPBi/LiF/Ag device with mixed large/small grains. We can see in Figure 6A that increasing an external bias can largely increase the low-frequency C-f signal amplitude (∼1 kHz). The low-frequency signal (at 1 kHz) is increased by 33% upon increasing the bias from 0 to 1.6 V toward the turn-on voltage (1.9 V). This provides an experimental indication that field-dependent surface polarization occurs in the perovskite MAPbBr_3_ with mixed large/small grains. On contrast, when only small grains are used in the MAPbBr_3_ film, increasing an external bias does not change the low-frequency capacitance signal, lacking the filed-induced surface polarization (Figure 6B). We should note that the electrical polarization consists of charged effects at grain boundaries and intrinsic bulk polarization given by

(Equation 2) P_surface_ = P_defects_ + P_bulk_

where *P*_*defects*_ and *P*_*bulk*_ represent charged defects and bulk polarization. When the *P*_*defects*_ is removed by self-passivation, the *P*_*bulk*_ can lead to a field-induced polarization on the perovskite surface. Clearly, the field-induced surface polarization can result in a lower turn-on bias than the bandgap, forming a necessary condition to develop high EL performance in perovskite LEDs. We should note that organic-inorganic hybrid perovskites possess bulk orientational/dipolar polarization (Frost et al., 2014), functioning as electrically polarizable semiconductors (Blom et al., 1994). However, the charged effects at grain boundaries can screen the bulk polarization. We observed that, when grain boundary defects are passivated by doping, the bulk polarization can be largely enhanced (Wu et al., 2017b). Here, we can see in Figure 6A that the self-passivation increases the field-dependent surface polarization and consequently leads to a turn-on voltage lower than the bandgap. Therefore, the self-passivation from electrically driven ion migration provides a unique mechanism to develop high-performance perovskite LEDs.

### Conclusion

In summary, we found that subsequently growing large and small grains at micro- and nano-scales presents a new method to develop high-performance perovskite LEDs through one-step solution processing by mixing two precursor solutions (PbBr_2_ + MABr and Pb(Ac)_2_·3H_2_O + MABr). Essentially, subsequently growing large and small grains is realized by using concurrently occurring fast and slow growths to form a unique configuration of attaching small grains to the surfaces of large grains, leading to mixed large/small grains in the perovskite (MAPbBr_3_) films by using our method. Interestingly, this method enables self-passivation of grain boundary defects, where electrically driven ions can physically passivate grain boundary defects, to develop high-performance perovskite (MAPbBr_3_) LEDs. Upon self-passivation during device operation, the EL shows the high brightness of 49,119 cd/m^2^ and stable efficiency of 54.6 cd/A without roll-off problem. Simultaneously, the EL output shows an angle dependence relative to the electric field direction, indicating that the light-emitting states become linearly polarized after self-passivation by the applied electric field. Furthermore, the self-passivation enables the field-dependent surface polarization, leading to a turn-on voltage lower than the bandgap to initiate the EL action. Therefore, subsequently growing large and small grains at micrometer and nanometer scales with the configuration of attaching small grains to the surfaces of large grains presents an important strategy to develop high-performance perovskite LEDs with polarized EL output, high brightness, stable efficiency, and low turn-on voltage.

### Limitations of the Study

Attaching small grains onto the surfaces of large grains is realized by mixing two precursor solutions by initiating concurrently occurring fast and slow crystallizations. Here, the PbBr_2_-based precursor solution (PbBr_2_ + MABr in DMF) results in cubic millimeter-sized large grains through fast crystallization and the Pb(Ac)_2_·3H_2_O-based precursor solution (Pb(Ac)_2_·3H_2_O + MABr in DMF) gives rise to small grains through slow crystallization. Essentially, mixing these two precursor solutions leads to the unique configuration whereby the small grains are attached to the surfaces of large grains to form perovskite (MAPbBr_3_) films with mixed large/small grains. By attaching small grains to the surfaces of large grains, the mobile ions can be electrically driven to the interfaces of large/small grains to passivate the defects during device operation, leading to a self-passivation of defects in perovskite LEDs prepared with mixed large/small grains. The key point is to attach small grains to the surfaces of large grains to form mixed large/small grains with low/high bandgaps in perovskite films.

## Methods

All methods can be found in the accompanying Transparent Methods supplemental file.

## Supplemental Information

Document S1. Transparent Methods and Figures S1–S4
