# Supplementary material for: Enabling Self-passivation by Attaching Small Grains on Surfaces of Large Grains toward High-Performance Perovskite LEDs
Source: iScience. 2019 Jul 31;19:378–87. doi: 10.1016/j.isci.2019.07.044 (PMC6706605; doi:10.1016/j.isci.2019.07.044)
Supplement: Document S1. Transparent Methods and Figures S1–S4 [file mmc1.pdf]

ISCI, Volume *19*

Supplemental Information

Enabling Self-passivation by Attaching Small Grains on Surfaces of Large Grains toward High-Performance Perovskite LEDs

Jiajun Qin, Jia Zhang, Yujie Bai, Shengbo Ma, Miaosheng Wang, Hengxing Xu, Matthew Loyd, Yiqiang Zhan, Xiaoyuan Hou, and Bin Hu

Enabling self-passivation by attaching small grains on surfaces of large grains towards high-performance perovskite LEDs

Jiajun Qin, Jia Zhang, Yujie Bai, Shengbo Ma, Miaosheng Wang, Hengxing Xu, Matthew Loyd, Yiqiang Zhan, Xiaoyuan Hou and Bin Hu

Supplemental figures

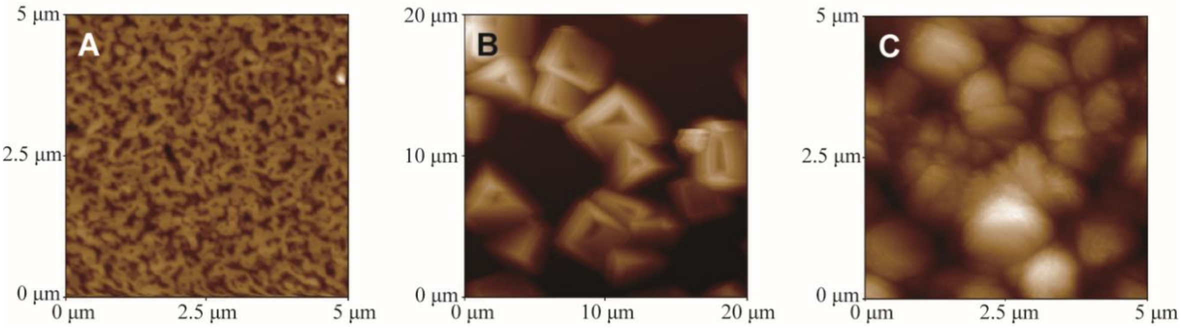

Figure S1. AFM images for (A) small grains-only film, (B) large grains-only film, (C) mixed large/small grains. Related to Figure 1.

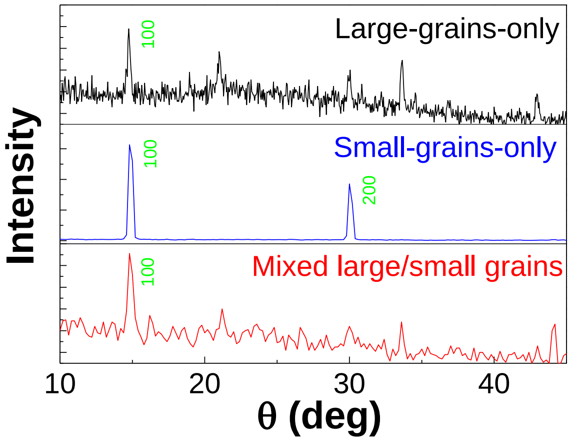

Figure S2. X-ray diffraction (XRD) patterns for three different perovskite film ($MAPbBr_3$). Related to Figure 1.

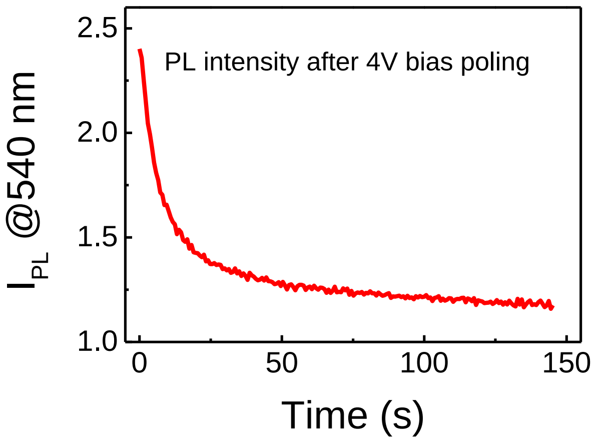

Figure S3. After 4V bias poling, with immediately applied photoexcitation (405 nm) PL is slowly decayed in perovskite LED with mixed large/small grains. Related to Figure 2.

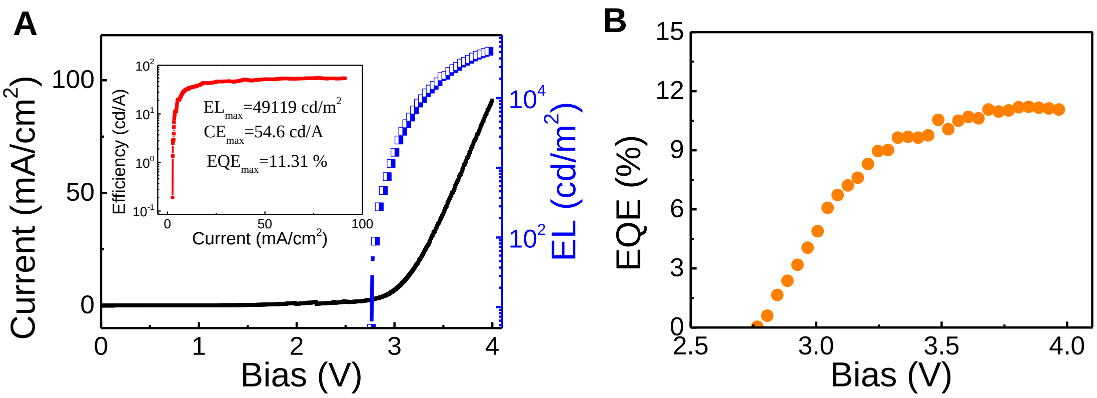

Figure S4. (A) EL-current-voltage characteristics of optimized device with the structure ITO/PEDOT:PSS/$MAPbBr_3$/Bphen(20 nm)/Bphen:PMMA(1:2 10mg/ml)/LiF(0.7 nm)/Ag. The scanning rate is 0.01 V/step with each step duration of 0.1 s. Such slow scan from 0 to 4 V is used to enable sufficient self-passivation of defects. (B) EQE-voltage characteristics. **Related to Figure 5.**

Transparent Methods

Materials processing and device Fabrication: The ITO substrates were first cleaned by ultrasonic treatment respectively with detergent, deionized water, acetone and isopropanol for 20 minutes in each cycle. The cleaned ITO substrates were exposed to UV ozone for 30 minutes and followed by the spin coating of PEDOT:PSS with the thickness of 40 nm. The PEDOT:PSS films coated on ITO substrates were thermally annealed at 150 °C for 0.5 h.

The perovskite ($MAPbBr_3$) films with mixed large/small grains were prepared base on the following procedures. Firstly, two precursor solutions were prepared: (i) 1 mol/L DMF solution (with molar ratio $Pb(Ac)_2{\cdot}3H_2O$: MABr = 1:3) for small grains, and (ii) 1.5 mol/L DMF solution (with molar ratio $PbBr_2$: MABr = 1:1.05) for large grains. Secondly, these two solutions with volume ratio of 1:4 (more $PbBr_2$ volume portion) were mixed to prepare mixed large/small grains. The mixed solution was spin-coated on PEDOT:PSS films in nitrogen atmosphere at the rate of 3000 rpm for 1 minute to form $MAPbBr_3$ films with mixed large/small grains, then annealed for 30 minutes at 60 °C.

The TPBi, LiF and Ag were thermally deposited under vacuum with the thicknesses of 50 nm, 0.7 nm and 80 nm, respectively. Finally, the perovskite light-emitting devices were prepared with the architecture of ITO/PEDOT:PSS/$MAPbBr_3$/TPBi(50nm)/LiF(0.7nm)/Ag. The ETL (Bphen:PMMA) layer was prepared by spin-coating at the rate of 4 krpm for 1 minute. For the solution preparation, 10 mg Bphen and 20 mg PMMA were dissolved in 1 mL chloroform. The fabricated perovskite light-emitting devices were encapsulated by UV curable epoxy for experimental measurements.

Characterizations and measurements: The transient and steady-state PL characteristics were measured by using Flouro Log III spectrometer with lifetime acquisition. The film morphologies were characterized by AFM (Veeco, diCaliber). The current-voltage characteristics were measured by Keithley 2400. The EL brightness was characterized by using power meter (ST86LA). The capacitance-frequency characteristics were recorded with alternating bias of 50 mV by using dielectric spectrometer (Agilent, 4294A). All measurements were performed in nitrogen atmosphere.
